# Comparison of Open Appendectomy and Laparoscopic Appendectomy in Perforated Appendicitis

**DOI:** 10.7759/cureus.5105

**Published:** 2019-07-09

**Authors:** Aamna Nazir, Sarosh Afzal Farooqi, Noman A Chaudhary, Hamza Waqar Bhatti, Mahnoor Waqar, Abdullah Sadiq

**Affiliations:** 1 Surgery, Holy Family Hospital, Rawalpindi, PAK; 2 Surgery, Rawalpindi Medical University, Rawalpindi, PAK

**Keywords:** open appendectomy, laparoscopic, appendectomy, perforated appendix

## Abstract

Introduction

Laparoscopic appendectomy for nonperforated appendicitis is associated with improved outcomes. This study compares laparoscopic appendectomy and open appendectomy in cases of a perforated appendix by assessing surgical site infection, mean operating time, and length of hospital stay.

Materials and methods

This study was a prospective randomized study conducted at the Department of Surgery, Holy Family Hospital, Rawalpindi, Pakistan, from January 2016 to January 2017, by randomly allotting the laparoscopic or the open appendectomy technique to 130 patients by the lottery method. Patients having a perforated appendix were included after they provided informed consent. Data were entered and analyzed using IBM SPSS Statistics for Windows, Version 20.0 (IBM Corp., Armonk, NY, US).

Results

The frequency of wound site infection was significantly higher in open appendectomy (27.69%) than in the laparoscopic approach (10.77%; p=0.01). Mean hospital stay was slightly longer in the laparoscopic approach (4.38 ± 1.09 days) than in open appendectomy (4.18 ± 0.77 days; p=0.23). Mean operating time for laparoscopic appendectomy and open appendectomy was 46.98 ± 2.99 minutes and 53.02 ± 2.88 minutes, respectively (p<0.000).

Conclusion

Laparoscopic appendectomy was associated with fewer surgical site infections and shorter mean operating time than an open appendectomy.

## Introduction

Appendicitis is the inflammation of the vermiform appendix [1]. Acute appendicitis is the most common abdominal emergency worldwide, and it is the most common cause of abdominal surgeries in all the age groups [2]. Appendicitis has an overall lifetime risk of 8.6% in men and 6.7% in women [2-3].

Of all the patients presenting with acute appendicitis, 13% to 20% have a perforated appendix [4]. Men have a greater risk of perforation of the appendix (18%) than do women (13%) [5]. Although the risk of perforation is eminent 24 hours after the appearance of the symptoms of appendicitis, the time course varies from case to case. There is a 20% risk of perforation of the appendix within 24 hours of the appearance of symptoms [6].

Since its description by McBurney, open appendectomy has become the procedure of choice for acute appendicitis [7-8]. The field of surgery has dramatically changed since the advent of laparoscopy [9]. Laparoscopic appendectomy was first introduced by Semm [2]. It has gained much popularity among surgeons because of the use of minimally invasive techniques, but some remain skeptical about its use instead of open appendectomy [8]. Those who criticize laparoscopic appendectomy cite the increased operative costs of using disposable instruments. Other criticisms of laparoscopic appendectomy target the increased operating time and increased incidence of intra-abdominal abscesses, particularly in cases of a perforated appendix [10-11]. Proponents of laparoscopic appendectomy claim the procedure yields improved wound healing, reduced postoperative pain, and earlier discharge from the hospital, with an earlier return to normal activities [8].

Furthermore, laparoscopy has the advantages of minimal incision, a better view of the peritoneal cavity, and safe exploration [12]. The feasibility and validity of the laparoscopic approach in complicated (i.e., perforated) appendix cases remain controversial, as it is associated with an increased incidence of intra-abdominal collection, but several other trials have statistically found that the laparoscopic approach is associated with fewer postoperative complications [13]. Due to the lack of randomized prospective studies, there is a gap in the literature about the comparison of laparoscopy and laparotomy in the management of perforated appendix. Laparoscopic management has now become the preferred mode of management because it can diagnose and remove the appendix at the same time [14].

The aim of this study is to compare the outcomes of laparoscopic and open approaches in perforated appendicitis. Although a study was done on the comparison of both techniques in uncomplicated appendicitis, no study has yet been conducted on the comparison of these techniques for the removal of a perforated appendix [15].

## Materials and methods

Our study was a prospective randomized study conducted at the Department of Surgery, Holy Family Hospital, Rawalpindi, Pakistan, from January 2016 to January 2017. A total of 130 patients were included in the study, with 65 patients in each group. All patients aged 15 to 50 years who presented with a perforated appendix and ultrasonographic evidence of free fluid in the abdomen were included in the study. Patients with a perforated appendix were defined as those presenting with pain in the right iliac fossa for one or two days, with a history and examination suggestive of a perforated appendix, having lower abdominal tenderness, tachycardia, and fever (>99°F). Those who had a total leukocyte count of 10,000 and above and those who had evidence of free fluid in the lower abdomen or pelvis on ultrasonogram were also included in the study. Patients who had simple, uncomplicated appendicitis and who had undergone any previous abdominal surgery were excluded from the study. Patients who were anesthetically unfit with American Society of Anesthesiologists (ASA) class three or above and those with any general contraindication to laparoscopic surgery like morbid obesity, respiratory insufficiency, or history of tuberculosis were also excluded. Patients fulfilling the inclusion criteria were included after providing informed consent. Cases were randomized by a lottery method prospectively to open and laparoscopic appendectomy groups. The perioperative and postoperative effects were assessed by the nurses. All the information was recorded on a predesigned proforma. Outcome variables were port site infection, length of hospitalization, and mean operative time.

The operating time in minutes was taken from the point of port insertion until appendix retrieval. The length of hospitalization in days, from the time of admission to the time of discharge, was also recorded. Port site infection was defined as the presence of signs of inflammation (erythema and discharge) on the fourth-day follow-up evaluation in the outpatient department after the surgery.

All the patients either undergoing open or laparoscopic surgery were given single doses of intravenous injections of metronidazole 400 mg and ceftriaxone 1 g perioperatively, and the same doses were continued for five days postoperatively. The open approach was made by a lower midline laparotomy, appendectomy was done, and the abdomen was washed with normal saline. The abdomen was closed. However, the skin was left open. Laparoscopic appendectomy was done by creating a pneumoperitoneum via the three-port technique. Appendectomy was done, and the appendix retrieved through a glove-made specimen bag to minimize spillage. The abdomen was washed with normal saline. A first intravenous injection of ketorolac 30 mg was given as a painkiller immediately after the surgery. The second injection was given eight hours later, and the third was administered 72 hours after surgery.

The collected data were entered and analyzed using IBM SPSS Statistics for Windows, Version 20.0 (IBM Corp., Armonk, NY, US). Qualitative variables like gender and infection were measured as frequencies and percentages. The quantitative variables like age, length of hospitalization, and operative time were measured as mean ± SD. Independent samples t-test was used to compare the length of hospital stay and operating time between two groups. Effect modifiers like age, gender, and ASA class were controlled by stratification. Post-stratification chi-square tests were applied for qualitative variables and the independent samples t-test for quantitative variables. A p-value of ≤0.05 was considered significant.

## Results

A total of 130 cases (65 in each group) fulfilling the inclusion criteria were included in the study. Of 130 patients, 65 (50%) were male patients and 65 (50%) were female patients.

The mean age was 32 ± 7 years in the laparoscopic appendectomy group and 34 ± 7 years in the open appendectomy group. Twenty-nine patients were in the 15 to 30 years age group (44.62%) in the laparoscopic surgery group, and 27 patients were aged 15 to 30 years in the open surgery group (41.54%). The laparoscopic surgery group had 36 patients aged 31 to 50 years (55.38%), and the open surgery group had 38 patients (58.46%) aged 31 to 50 years.

Patients were almost equally distributed according to gender in both groups. The laparoscopic surgery group contained 33 male patients (50.77%) and 32 female patients (49.23%). The open surgery group contained 32 male patients (49.23%) and 33 female patients (50.77%).

In comparing the mean operating time in both groups, the mean operating time for the laparoscopic surgery group was 46.98 ± 2.99 minutes, which was significantly shorter than the 53.02 ± 2.88 minutes from the open surgery group (p<0.000). The mean length of hospitalization was 4.38 ± 1.09 days in laparoscopic surgery and 4.18 ± 0.77 days in the open surgery group (p=0.23). Seven port sites (10.77%) in the laparoscopic group and 18 (27.69%) in the open surgery group were infected (p= 0.01). The comparison of mean operating time, length of hospitalization, and rate of surgical site infections are shown in Table 1.

**Table 1:** Comparison of operating time and length of hospitalization in laparoscopic and open appendectomy.

Outcome Variable	Laparoscopic Appendectomy	Open Appendectomy	p-value
Operating time (mean ± SD)	46.98 ± 2.99 minutes	53.02 ± 2.88 minutes	<0.000
Length of hospitalization (mean ± SD)	4.38 ± 1.09 days	4.18 ± 0.77 days	0.23
Rate of surgical site infections (frequency (%))	7 (10.77%)	18 (27.69%)	0.01

Effect modifiers like age, gender, and ASA grades were controlled by stratification. The results of post-stratification chi-square tests (for qualitative variables) and independent samples t-tests (for quantitative variables) are shown in Table 2.

**Table 2:** Stratification for operation time, length of hospital stay, and wound infection with regards to age, gender, and ASA class. ASA, American Society of Anesthesiologists.

Dependent Variables (Outcome Variables)	Independent Variables (Explanatory Variables)	Groups	Laparoscopic Appendectomy	Open Appendectomy	p-value
Operation time (minutes, mean±SD)	Age	15-30 years	47.14 ± 3.10	53.19 ± 2.99	0.0001
		31-50 years	46.86 ± 2.94	52.89 ± 2.84	0.0001
	Gender	Male	47.09 ± 3.16	53.13 ± 2.89	0.0001
		Female	46.88 ± 2.88	52.91 ± 2.91	0.0001
	ASA	ASA-I	47.04 ± 3.36	53.25 ± 2.49	0.0001
		ASA-II	46.88 ± 2.86	53.02 ± 2.88	0.0001
Hospital stay (days, mean±SD)	Age	15-30 years	4.45 ± 1.12	4.22 ± 0.75	0.38
		31-50 years	4.33 ± 1.10	4.16 ± 0.79	0.0001
	Gender	Male	4.33 ± 1.14	4.09 ± 0.73	0.31
		Female	4.44 ± 1.08	4.27 ± 0.80	0.48
	ASA	ASA-I	4.32 ± 1.01	4.25 ± 0.78	0.74
		ASA-II	4.50 ± 1.25	4.08 ± 0.76	0.16
Wound infection (frequency (%))	Age	15-30 years	3/29 (10.34)	6/27 (22.22)	0.19
		31-50 years	4/36 (11.11)	12/38 (31.58)	0.03
	Gender	Male	4/33 (12.12)	8/32 (25)	0.18
		Female	3/32 (9.37)	10/33 (30.30)	0.03
	ASA	ASA-I	5/41 (12.19)	9/40 (22.5)	0.22
		ASA-II	2/24 (8.33)	9/25 (36)	0.02

## Discussion

Laparoscopy has been considered a relative contraindication in complicated appendicitis, as it is associated with an increased risk of postoperative complications [16-18]. This theory has been challenged by the findings of several studies that measured the outcomes of laparoscopic appendectomy in complicated appendicitis cases [19-21].

Muhammad et al. conducted a similar study and reported that the mean age in the laparoscopic appendectomy group was 32 ± 14 years; the mean age of patients in the open appendectomy group was 34 ± 13 years [12]. These results are quite close to the mean ages in our study. This similarity in age is because appendicitis is more common in the younger age group, as shown by Thomas et al. [22]. According to Drinkovic et al., appendicitis was most common in the 11 to 20-year age group, but the increasing incidence in older patients may be due to increased life expectancies [23-24].

The significantly shorter mean operating time for laparoscopic as compared to open appendectomy noted in our study differs from Muhammad et al.’s findings, who reported the mean operating time as 75 ± 23 minutes for a laparoscopic appendectomy and 64 ± 15 minutes for an open appendectomy [12]. Another study conducted by Lin et al. showed that laparoscopic appendectomy took a longer time to complete (96.1 ± 43.1 minutes) than open appendectomy (67.8 ± 32.2 minutes) [14]. Additional studies suggest the laparoscopic approach is associated with longer operating times than an open appendectomy [25-28]. These results were in contradiction to ours. However, our findings of shorter mean operating times via the laparoscopic approach align with studies by Yau et al. and Tiwari et al., who found a mean operating time for laparoscopic appendectomy were 47.8 ± 14.5 minutes and 49.10 ± 12.5 for open appendectomy [13,29]. The variation reported in the literature in mean operating times may be due to variations in skill levels and experience with laparoscopic techniques in different centers.

Comparison of mean hospital stay in both groups in our setup showed an insignificant difference between the laparoscopic appendectomy group (4.38 ± 1.09 days) and the open appendectomy group (4.18 ± 0.77 days). However, Muhammad et al. reported the mean length of hospitalization for the laparoscopic appendectomy group was 5.3 ± 2.1 days while open appendectomy group had a mean length of hospitalization of 7.2 ± 3.2 days [12]. Tiwari et al. reported a significant difference in the length of hospital stay between groups (4.34 ± 4.84 days in the laparoscopic appendectomy group, 7.31 ± 9.34 days in the open appendectomy group) [13]. Lin et al. also reported that the length of hospital stay was significantly shorter for laparoscopic appendectomy (6.3 ± 2.9 days) than that for open appendectomy (9.3 ± 8.6 days) [14].

Our port site infection comparison yielded findings similar Muhammad et al., who reported that the rate of infections in the laparoscopic appendectomy group was 8.3% while that in the open appendectomy group was 24.4% [12]. Lin et al. also showed that the rate of infections was significantly lower in laparoscopic appendectomy (15.2%) than in open appendectomy (30.7%) [14]. This may be attributed to the fact that laparoscopic appendectomy requires less manipulation of the gut by the surgeon’s hands and instruments as compared to open appendectomy. Furthermore, the gut does not come into contact with the incision in the layers of the anterior abdominal wall during laparoscopic appendectomy as the appendix is explored in situ.

The results of post-stratification chi-square tests revealed that the operating time for the laparoscopic and open appendectomy was significantly different in the 15 to 30-year age group than in the 31 to 50-year group. The operating time was also significantly different for the two techniques in both male patients and female patients and ASA classes one and two. The difference in the length of hospital stay was also statistically significant between the two techniques for the 31 to 50-year age group. This might be due to the postoperative complications associated with the older age group. Wound infections were significantly more frequent in open appendectomy in the older age group as well. Infections were also significantly more frequent in female patients and in ASA class two for the open appendectomy. These factors might contribute to decreased immunity and increased risk of infections in these groups.

## Conclusions

Laparoscopic appendectomy is superior to open appendectomy in terms of wound site infections and operating time. The operating time depends on the surgical skills of the operating surgeon and the magnitude of the condition. With regards to the length of hospital stay, there is no difference between the two techniques. Thus, the laparoscopic appendectomy can be safely adopted for the removal of the perforated appendix.
